# Modelling to inform prophylaxis regimens to prevent human rabies

**DOI:** 10.1016/j.vaccine.2018.11.010

**Published:** 2018-12-07

**Authors:** Katie Hampson, Bernadette Abela-Ridder, Omesh Bharti, Lea Knopf, Monique Léchenne, Rolande Mindekem, Arnaud Tarantola, Jakob Zinsstag, Caroline Trotter

**Affiliations:** a Institute of Biodiversity, Animal Health & Comparative Medicine, University of Glasgow, Glasgow G12 8QQ, UK; b Department of the Control of Neglected Tropical Diseases, 1121 Geneva 27, Switzerland; c State Institute of Health and Family Welfare, Himachal Pradesh, India; d Swiss Tropical & Public Health Institute, PO Box, 4002 Basel, Switzerland, University of Basel, Petersplatz 1, 4003 Basel, Switzerland; e Centre de Support en Sante International (CSSI), N’Djamena, Chad; f Epidemiology & Public Health Unit, Institut Pasteur du Cambodge, Phnom Penh, Cambodia; g Disease Dynamics Unit, Department of Veterinary Medicine, University of Cambridge, Madingley Road, Cambridge CB3 0ES, UK

**Keywords:** Post-exposure prophylaxis, Pre-exposure prophylaxis Expanded program on immunization, Rabies immunoglobulin, Intradermal, Intramuscular, Regimen, Dose-sparing

## Abstract

**Background:**

The Strategic Advisory Group of Experts (SAGE) Working Group on rabies vaccines and immunoglobulins was established in 2016 to develop practical and feasible recommendations for prevention of human rabies. To support the SAGE agenda we developed models to compare the relative costs and potential benefits of rabies prevention strategies.

**Methods:**

We examined Post-Exposure Prophylaxis (PEP) regimens, protocols for administration of Rabies Immunoglobulin (RIG) and inclusion of rabies Pre-Exposure Prophylaxis (PrEP) within the Expanded Programme on Immunization (EPI). For different PEP regimens, clinic throughputs and consumables for vaccine administration, we evaluated the cost per patient treated, costs to patients and potential to treat more patients given limited vaccine availability.

**Results:**

We found that intradermal (ID) vaccination reduces the volume of vaccine used in all settings, is less costly and has potential to mitigate vaccine shortages. Specifically, the abridged 1-week 2-site ID regimen was the most cost-effective PEP regimen, even in settings with low numbers of bite patients presenting to clinics. We found advantages of administering RIG to the wound(s) only, using considerably less product than when the remaining dose is injected intramuscularly distant to the wound(s). We found that PrEP as part of the EPI programme would be substantially more expensive than use of PEP and dog vaccination in prevention of human rabies.

**Conclusions:**

These modeling insights inform WHO recommendations for use of human rabies vaccines and biologicals. Specifically, the 1-week 2-site ID regimen is recommended as it is less costly and treats many more patients when vaccine is in short supply. If available, RIG should be administered at the wound only. PrEP is highly unlikely to be an efficient use of resources and should therefore only be considered in extreme circumstances, where the incidence of rabies exposures is extremely high.

## 1 Background

Considerable efforts are underway to reduce the burden of rabies, with the goal of reaching zero human deaths due to dogmediated rabies by 2030 [1]. The main strategies to prevent human rabies are (1) canine vaccination to eliminate rabies at its source and (2) Post-Exposure Prophylaxis (PEP) comprising wound washing, vaccination and, when indicated, Rabies Immunoglobulin (RIG), to individuals bitten by suspect rabid animals [2]. An additional preventive strategy is Pre-Exposure Prophylaxis (PrEP) whereby vaccine is given to prime the immune system [3]. Individuals who have received rabies PrEP still require PEP, but need fewer (booster) doses than unprimed individuals and do not require RIG. To support the development of practical and feasible recommendations for human rabies prevention WHO established a Strategic Advisory Group of Experts (SAGE) Working Group on rabies vaccines and immunoglobulins in 2016 (http://www.who.int/immunization/policy/sage/sage_wg_rabies_jul2016/en/). As part of this process, we compared PEP regimens, approaches to RIG administration and PrEP to assess their relative merits.

PEP is extremely effective if administered promptly to exposed individuals and several regimens have been recommended [4]. Intradermal (ID) regimens are more cost-effective than intramuscular (IM) regimens [5], because smaller volumes of vaccine are used to elicit a clinically equivalent immune response. So far only a few countries have adopted ID vaccination, with several factors likely contributing. Fractionated vials should be discarded within 6–8 h to minimize risks of bacterial contamination (rabies vaccines do not contain preservatives) [4,6], which is often perceived as waste [1]. Inexperienced clinicians may consider ID vaccination to require more skill, and fear that smaller doses are less protective. Rabies vaccine is available as 0.5 mL or 1 mL vials. Using standard syringes with mounted needles, clinicians usually obtain four ID doses of 0.1 mL from 0.5 mL vials and 8 from 1 mL vials, with wastage of 20%. Use of more expensive insulin syringes with built-in needles and no dead space prevents such wastage and the same needle can be used to withdraw vaccine and inject the patient.

RIG is recommended for severe exposures to potentially rabid animals and all bat exposures, providing passive immunity while the vaccine elicits an active immune response [7]. The previous WHO recommendation calculates dosage according to patient body weight, with as much RIG as is anatomically possible being administered into and around the wound(s) and the remaining product being injected intramuscularly distant from the wound [4]. New evidence led to revised protocols for administering RIG at the wound only [8,9]. RIG vials can be shared between patients using single-use injection devices, but opened vials should also be discarded at the end of each day.

A systematic review on safety, immunogenicity, costeffectiveness and recommendations for use of rabies PrEP concluded that PrEP “is safe and immunogenic and should be considered: (i) where access to PEP is limited or delayed; (ii) where the risk of exposure is high and may go unrecognized; and (iii) where controlling rabies in the animal reservoir is difficult” [3]. Rabies PrEP programmes have been implemented in Peru (for exposures via vampire bats) and the Philippines (targeting children at risk of dog-transmitted rabies) [3]. Offering widespread PrEP, for example within routine EPI programmes in rabies-endemic countries, raises practical and operational difficulties, as delivering multiple doses within short time scales (i.e. 1-week) lies outside the standard programme. However, if PrEP could be a costeffective method to prevent human rabies, ways to overcome these challenges should be considered.

We developed models to quantitatively assess the potential benefits and costs of these strategies for prevention of dogmediated rabies.

## 2 Methods

### 2.1 PEP regimens

We updated a simulation to compare PEP regimens [5] (Table 1). Briefly, our algorithm involved: (1) assigning patient presentation dates uniformly based on clinic throughput; (2) generating patient return dates based on specified regimens and patient compliance; (3) calculating daily vial use; (4) iterating steps 1–3 to capture variation.

Direct (medical) costs for vaccines and their administration and indirect (non-medical) costs (including transport to/from clinics) were taken from published data and expert consultation (Table 2). We assumed that vaccine administration time is equivalent for all regimens. For this analysis we did not include RIG because it is rarely available to bite victims in endemic countries [10–14].

We explored vial use according to:

*Clinic throughput:* monthly numbers of bite patients presenting to clinics to initiate PEP. Total presentations depend on the regimen, its schedule requirements (Table 1), clinic accessibility [15] and patient compliance.
*Vial size:* most rabies vaccines are sold in 0.5 mL or 1 mL vials, at equal cost. Vial size affects numbers of doses that can be withdrawn, as does syringe type.
*Vaccine wastage:* vaccine from opened vials must be used within 6–8 h or discarded. We assumed use of WHO pre-qualified rabies vaccines, with 0.1 mL doses for all regimens.
*Syringe type:* We compared costs of using insulin syringes that reduce waste compared to standard syringes (20% wastage). For all regimens we assumed use of an additional syringe per vial to reconstitute the vaccine.
*Patient compliance:* the probability of a bite patient completing PEP vaccination(s). Whatever its cause, poor compliance has consequences for vaccine use, vial sharing and PEP efficacy. We did not consider variability in return dates.

We ran 1000 realisations for each scenario to capture variation in patient presentation dates and vial sharing. We compared costs for bite victims depending upon pricing strategies and indirect costs (Table 2). We assume bite victims travel further to reach rural clinics compared to urban clinics and incur correspondingly higher costs, spanning the range from $2.5 to $15 per clinic visit [16]. To investigate limited vaccine supply we assessed the maximum number of patients that could be treated with a given volume of vaccine under different regimens.

### 2.2 RIG delivery

We undertook a similar analysis for RIG using data collected over 12-months from Himachal Pradesh, India. Due to limited RIG availability, patients (N = 700, median age: 30 years, median weight: 53 kg, sex ratio: 63:37 male:female) were administered RIG under a dose-sparing approach of infiltration of the wound only. All survived on follow-up [8,9]. We used bootstrap sampling of these data, where patient weight was measured, to capture variability in RIG use under two scenarios: (1) infiltration at the wound (s) with the remainder administered intramuscularly distant from the wound; and (2) infiltration of the wound(s) only. We assumed opened RIG vials were discarded at the end of each day and examined a range of clinic throughputs using 5 mL ERIG vials containing not less than 300 IU/mL, as available in Himachal Pradesh.

### 2.3 PrEP

We took two approaches to quantify the potential benefits and relative costs of including rabies PrEP within a routine EPI schedule in endemic settings:

#### a Hypothetical birth cohort

We developed a simple simulation model to estimate the relative cost of PrEP plus PEP versus PEP alone. This cost ratio largely depends on the incidence of dog bites (for which individuals seek PEP) and the cost per course of PrEP plus PEP vs PEP alone.

*Bite incidence*: The incidence of dog bites in endemic settings has been reported to vary from around 12 per 100,000 population (Chad) to around 1200 per 100,000 population (India, Sri Lanka, Cambodia, Myanmar) [17]. A more recent systematic review covering 2013–2015 reported typical bite incidence in the range 10–130 per 100,000 per year (WHO, unpublished). The highest reported bite incidence we identified in the literature is 4840 per 100,000 in rural Cambodia which is far higher than reported in any other setting [18]. Since PEP costs are only relevant for individuals who seek care, crude bite incidence should be modified by the proportion of people seeking care. We modelled a typical range of care-seeking bite victims of 10–500 per 100,000 population per year.

*Costs of PrEP and PEP*: We did not model all regimens considered for PEP and PrEP individually, assuming any differences in rabies prevention would be marginal. We assumed costs of PEP for those who have been primed to be the same as for PrEP, but this does not fully capture the variation in all of the recommended regimens. Although it is the relative rather than absolute costs that are important, for simplicity we assumed that dose-sparing regimens would be used giving PrEP costs of between $5 and $20 and PEP costs in naïve individuals of $10–160, with the upper end of this range high enough to include RIG provision. We did not consider tetanus vaccination which is often given alongside PEP.

*Simulation*. Assuming that both bite incidence and costs of PrEP and PEP followed a uniform distribution we ran 10,000 simulations to estimate the ratio of costs for a hypothetical cohort of 100,000 children with high EPI vaccine uptake. We assumed protection from PrEP lasted for 20 years as an optimistic assumption based on limited data [3] and that dog bites are most common in children. Future costs were not discounted.

#### b N’Djamena, Chad

A model was previously developed to estimate the cost-effectiveness of PEP alone to prevent human rabies deaths versus PEP plus dog vaccination for N’Djaména, Chad [14]. To evaluate the cost-effectiveness of PrEP in the same setting, we compared a scenario incorporating PrEP vaccination of a yearly cohort of children with these published scenarios. For all scenarios we assumed maximum access to PEP and that communication between the veterinary and human health sector guided treatment.

The number of suspected rabies exposures were taken from data collected in 2012 in approximately 30% of all health facilities in N’Djaména, including all public health centres and hospitals, prior to a mass dog vaccination campaign [19]. Suspicion was defined based on the animal status and was attributed to bites from unvaccinated animals and animals that died, vanished, or were killed without laboratory diagnosis within 10 days of the bite. The Essen 5-dose IM regimen is used in N’Djaména with PEP costing 198 USD including transport and personnel, but not RIG, which is unavailable in Chad. We considered PrEP costs of 83 USD covering 3 vaccine doses, transport, loss of work time and personnel for administration. We assumed that pre-vaccinated children require 2 additional vaccine doses if exposed, amounting to 66.5 USD.

We assumed PrEP coverage of 55% based on observed ‘measles 1’ vaccination coverage in Chad [20]. This is probably optimistic as a full rabies PrEP schedule requires 2 visits [4] rather than just 1 for measles. To achieve this coverage, approximately 57,270 children must be vaccinated annually in N’Djaména. We simulated the change of PrEP coverage in children under 15 years over a 20-year period using a demographic model with data from the 2009 national census. Multiplying this coverage by the percentage of children among exposure victims generated an increasing number of children requiring two PEP doses instead of 5. The overall cost of this scenario is the sum of costs for PrEP, PEP for pre-vaccinated children and PEP for unvaccinated children and adults. Cumulative costs were discounted at a rate of 0.04 [8] and DALYs averted were based on a 19% risk of developing rabies after exposure by a suspect rabid animal [21].

Analyses were conducted using R version 3.4.1 except for the N’Djamena demographic model which was conducted in vensim. Code and data for reproducing the PEP and RIG simulations are available at: https://github.com/katiehampson1978/Modelling_rabies_regimens

## 3 Results

### 3.1 PEP regimens

**Fig. 1:**
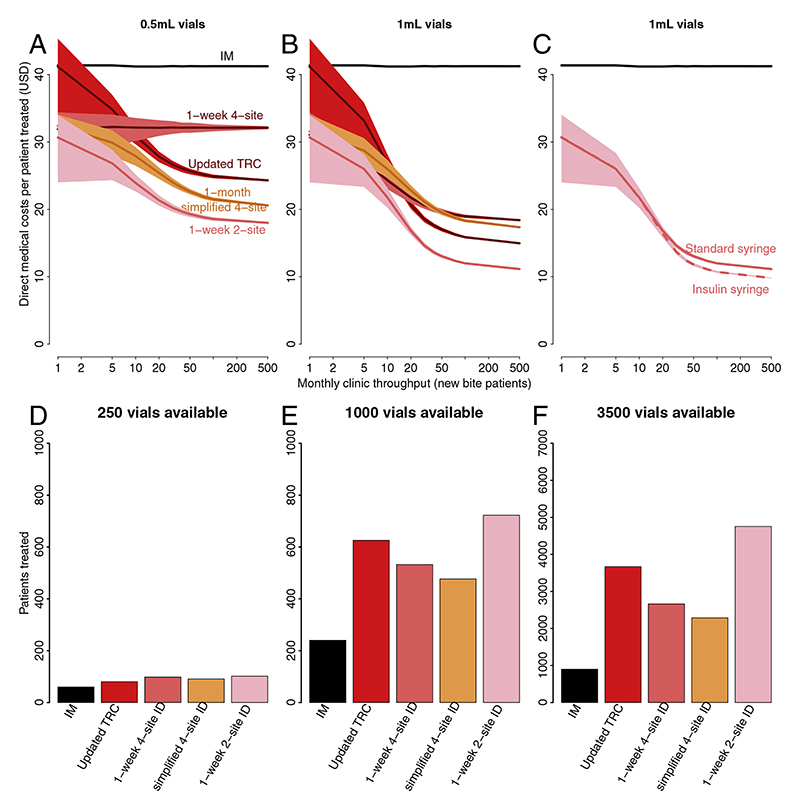
Comparison showing that ID PEP regimens are more cost-effective per patient treated and able to treat more patients given limited vaccine availability. In panels (A–C) direct medical costs per patient treated are shown in relation to clinic throughput, vial size and syringe type. The cost of both approved IM regimens are equivalent (solid black line) and do not change with clinic throughput whereas the cost of ID regimens improves with patient throughput as vials can be shared between patients using single-use injection devices - with greater savings for equivalently priced 1 mL vs 0.5 mL vials, especially in high-throughput clinics. Only the most cost-effective regimen, the 1-week 2-site ID regimen, is shown in panel C comparing insulin versus standard syringes. In panels (D–F) we assumed clinics only had only 250, 1000 or 3500 vials available over a 1-year period. Note the different y-axis limits for panel F.

**Fig. 2:**
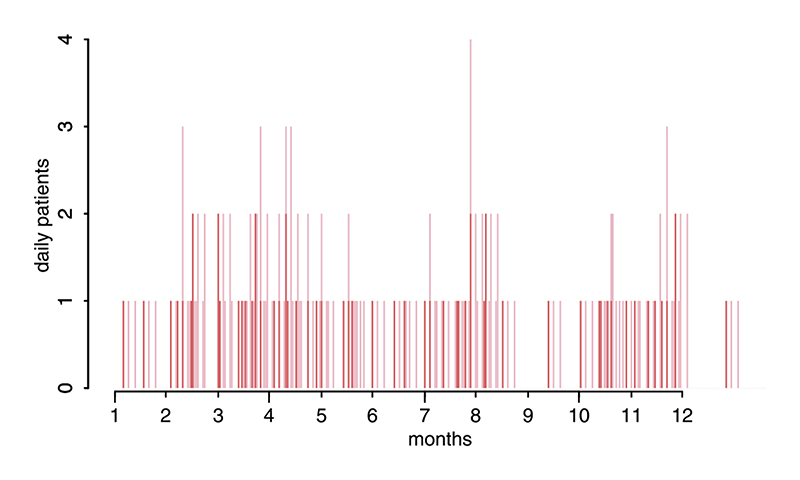
Example patient presentation dates in a low throughput clinic with an average of 5 new patients presenting monthly. Red bars show dates patients initiated PEP and pink bars correspond to subsequent return dates following the 1-week 2-site ID regimen. In this example 140 vials are used with vials shared on days with 2 or more patient presentations. In this situation because of vial sharing 20% fewer vials are used than if no vials were shared. (For interpretation of the references to colour in this figure legend, the reader is referred to the web version of this article.)

Overall, ID vaccination is always more cost-effective than IM vaccination, is less costly for health providers and either costs less or equivalent for patients (Fig. 1, Table 1). The cost-effectiveness of IM vaccination does not change with clinic throughput. For ID vaccination cost-effectiveness increased with throughput (and if reconstituted vaccine could be stored for longer than 8 h costeffectiveness would further increase at lower throughputs). The 1-week 2-site ID regimen is the most cost-effective regimen in all settings because less vials are used per course than for IM vaccination (4 vs 3 with 1 mL vials), even without vial sharing. The requirement for patients to return to clinics for subsequent doses means opportunities for vial sharing occur even in low throughput settings (Figs. 2 and S1). In high-throughput clinics the 1-week 2-site ID regimen uses 85% fewer 1 mL vials than IM regimens (Fig. 1). Use of insulin syringes rather than standard syringes with mounted needles further reduced costs, particularly in clinics receiving >10 new bite patients each month (Fig. 1). The same qualitative patterns were observed with lower patient compliance, therefore these results are not presented.

ID regimens are dose-sparing and have greater potential to treat more patients given limited vaccine supply (Fig. 1). The 1-week 2-site ID regimen can treat 5 times as many patients than IM regimens given an annual supply of 3000 vials (>70 new patients per month).

Where PEP is provided free-of-charge, the 3-week IM and 1-week ID regimens are preferable for patients, who incur only indirect costs, as both require just 3 clinic visits (Table 1). When patients must pay for PEP, the most preferable regimen depends on pricing strategies and relative travel costs, but ID regimens are always preferable to IM.

### 3.2 RIG delivery

**Fig. 3:**
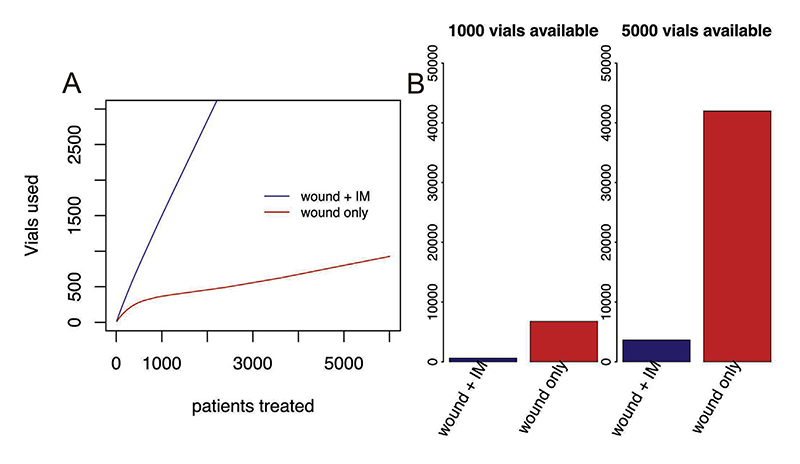
Comparison of patients treated with RIG when infiltrated at the wound with the remainder administered intramuscularly distant from the wound (blue) and when infiltrated to the wound(s) only (red). (A) Vials used under different patient throughputs and (B) patients treated given limited vial availability with examples shown for 1000 vials and 5000 vials per year.

Infiltration of RIG at the wound(s) only results in considerable savings, that increase with patient throughput as vials can be more effectively shared between patients using single-use injection devices (Fig. 3). Moreover, when available vials are limited, many more patients can be treated if RIG is only administered at the wound site(s). In the clinic in Himachal Pradesh, around 270 patients are seen per month requiring approximately 262 RIG vials if injected at the wound only, versus 370 vials if the remainder is administered distant to the wound, a 40% reduction.

### 3.3 PrEP in the routine EPI schedule

#### a Hypothetical birth cohort

**Fig. 4:**
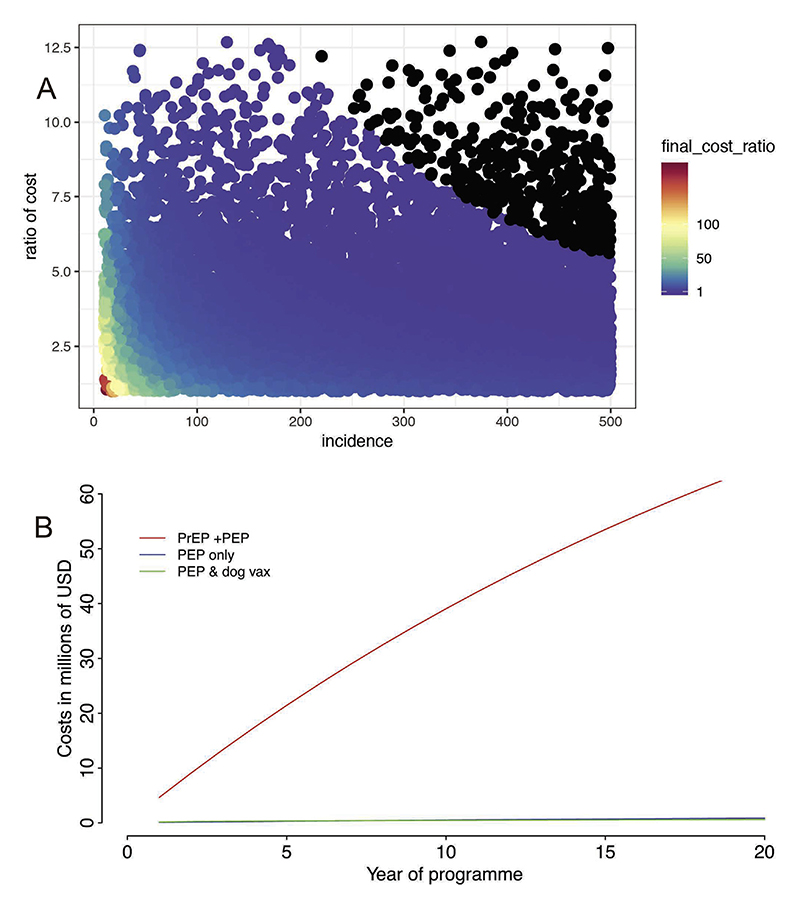
Comparison of the costs of rabies prevention using PrEP plus PEP versus PEP or in combination with dog vaccination. In (A) the costs of PrEP plus PEP versus PEP alone is shown in relation to dog bite incidence in a generic cohort model and in (B) extrapolated cumulative costs of PrEP plus PEP, PEP alone, and PEP and dog vaccination are shown for N’Djamena, Chad. In (A) simulations with a final cost ratio ≤1 are indicated in black.

Use of PrEP plus PEP boosters was at least twice as expensive as PEP alone in 75% of simulations. In some simulations where bite incidence was low and PEP costs in naïve individuals were also relatively low, the ratio was in the range of 100–200. In 4% of simulations (Fig. 4, black points) the ratio was ≤1, meaning that PrEP plus PEP was less expensive than PEP alone; here both bite incidence and relative PEP costs in naïve individuals were very high.

#### b N’Djamena, Chad

The annual number of suspect rabies exposures for N’Djamena based on reported bite patients was 374, of which 42% were children [14]. This is a conservative estimate, since the survey did not cover all pharmacies and private medical facilities. We estimate that after 20 years the cumulative cost of PrEP plus PEP was over fifty times higher than the cumulative cost of PEP alone and PEP with mass dog vaccination (Fig. 4). The cost per DALY averted is $3242 USD versus just $43 USD using only PEP. In practice, 100% access to PEP is impossible to achieve, but even when compared to observed PEP use in N’Djamena [14], which prevents rabies at a cost of 171 USD per DALY averted, PrEP remains much less cost-effective. PrEP coverage among children stabilizes at around 35% after 35 years, whereas coverage in adults stabilizes at 35% only after 40 years.

## 4 Discussion

The models we developed provide evidence to support practical and feasible recommendations for human rabies prevention in different clinical settings. The safety and efficacy of ID administration of rabies PEP has been recognized for decades [33]. New data supports the clinical efficacy of an abridged ID regimen [28,34], and confirms that ID vaccination can be safely completed within one week [35]. Our results further establish that ID vaccination could considerably reduce vial use, mitigate shortages and provide more equitable access by making PEP more affordable. Specifically, the 1-week 2-site ID regimen was the most cost-effective regimen, with further advantages of enabling PEP to be completed within 1 week, requiring only 3 clinic visits and treating more patients when vaccine is in short supply. We found similar advantages of RIG administration to the wound(s) only, an advantage that is more pronounced in higher throughput clinics. We found that PrEP would be substantially more expensive than other measures to prevent human rabies deaths, such as PEP and mass dog vaccination in almost all settings.

Clinic throughput affects capacity for vial sharing, and therefore the cost-effectiveness of ID versus IM vaccination. But, ID regimens always use at least 25% fewer vials than IM, and as throughput increases, ID regimens become increasingly cost-effective, using up to 85% fewer vials. ID vaccination is safe, well-tolerated and clinically efficacious [4]. Health workers routinely deliver BCG immunizations intradermally [36], so there should be no technical difficulty in switching to ID administration. Use of insulin syringes should further reassure clinicians and reduce wastage as more accurate vaccine volumes can be injected (Fig. S1). Single-use prefilled injection devices could eliminate vaccine waste and would be more user-friendly therefore should be considered for future development if costs can be kept low. Similarly, research into vaccine preservation could enable more economical vaccine use [37]. However, most critically, only two human rabies vaccines are currently WHO prequalified [2]. Prequalification is an important step to accelerate widespread adoption of the new abridged 1-week 2-site ID regimen and should be encouraged.

Our model has several simplifications: we assume that the day of the week does not affect the likelihood of presenting for PEP. But patients may be less likely to present on Sundays (when clinics are often closed) and/or more likely to present on Mondays or other days (e.g. after pay day), which may affect vial sharing. We also only consider a range of travel costs to clinics but in practice the location of clinics and transport access would affect indirect costs and delays to PEP provision. Moreover, we do not consider clustering of presentations as frequently occurs due to the same dog biting multiple people [38–41], which increases opportunities for vial sharing. Finally, an opened vial may be additionally used for PrEP in non-bitten family members or individuals whose occupation increases their risk of exposure.

We also find major advantages of RIG administration to the wound site(s) only. This allows many more patients to be treated given limited RIG availability, an advantage that is more pronounced in high throughput settings (Fig. 3). This analysis assumes patients comparable to those from Himachal Pradesh in terms of body weight and wounds. Preservatives could enable opened RIG vials to be safely stored at 2–8 °C for up to 28 days increasing vial sharing opportunities, which should be further investigated.

Our results showed that mass pre-exposure vaccination for rabies is unlikely to be an efficient use of scarce resources. We assume PEP will always be necessary after exposure to a potentially rabid animal, therefore there are limited health benefits and substantial costs associated with PrEP. Use of PrEP in the EPI schedule targets many more children than are likely to be exposed to rabies and unlike most other infectious diseases, this risk is identifiable (i.e. bite victims can be targeted for PEP [40,41]). Of course, this assumes that PEP is available, which may not always be the case. However, in view of vaccine shortages observed in some countries, diverting vaccine from PEP to PrEP could be fatal for exposed victims and marginalized communities that already have limited PEP access and would likely also have limited PrEP access, compounding health inequalities. Any country seriously considering routine PrEP should assess relative effectiveness and cost-effectiveness of this approach compared to other measures, using models informed by local epidemiology.

We used two modelling approaches, one generic and one context-specific, to address the potential costs and cost-effectiveness of PrEP. For the hypothetical birth cohort we simulated a range of bite incidence and relative costs. We did not take into account differential immunogenicity as all regimens are expected to be clinically equivalent, nor did we account for age-specific variation in dog bites. In the specific example, parameters were based upon extensive field studies. We did not model PrEP use in other settings but there are additional studies reported in the literature (summarised in [3]). One from Thailand similarly found that bite incidence would need to be much higher than has been observed to make PrEP cost-comparable [42].

These analyses suggest that investing in PEP, and/or mass dog vaccination, will be preferable to PrEP. Our findings are in agreement with the recent systematic review of PrEP [3]. Even if the price of rabies vaccine were considerably lower, the marginal cost-effectiveness of PrEP is still likely to be less favourable than PEP or dog vaccination, simply because many more individuals need to be targeted.

## 5 Conclusions

Overall, we find that ID is more economical than IM vaccination. The 1-week 2-site ID regimen is the most cost-effective and could enable many more bite victims to be equitably treated with the same volume of vaccine. We recommend use of insulin syringes (Fig. S1) to efficiently administer ID vaccination and reassure clinicians. Moreover, we encourage further efforts to prequalify rabies vaccines to accelerate adoption of ID vaccination. Where RIG is available, it could be delivered to most patients by infiltration at the wound only. We find that PrEP as part of the EPI programme is highly unlikely to be an efficient use of resources and should only be considered in extreme circumstances, where incidence of rabies exposures is high in populations which cannot access timely PEP. Modelling could be used to support decision making in specific high-exposure contexts.

## Supplementary Material

Supplementary data to this article can be found online at https://doi.org/10.1016/j.vaccine.2018.11.010.

Fig S1

## Figures and Tables

**Table 1:** Rabies post-exposure vaccination regimens investigated and costs to patients under different pricing strategies and travel costs. The 5-visit Essen [22] and 5-visit TRC regimens [23] were not examined as these were previously replaced by the 2-week IM and 1-month 2-site ID regimens respectively [2]. Regimens included in the analysis were reviewed by the SAGE working group and deemed ± safe, but not WHO-approved/selected due to cost-effectiveness, feasibility or limited availability of clinical outcome data. We compared indirect costs assuming low travel costs ($2.5/clinic visit) for patients who live close to a clinic and high travel costs ($15/clinic visit) for those who live far from a clinic. Regimens with reduced numbers of patient visits to clinics have lower indirect costs, therefore reducing visits should be prioritized for future regimens.

Regimen	Route	Visits	Schedule (days)	Injections	Vials	Vol (mL)*	Travel		$2.5 ID dose/$10 vial		$15 PEP/$10 vial	
							Near	Far	Near	Far	Near	Far
2-Week IM (4-dose Essen) [24]	IM	4	0,3,7,14	1,1,1,1	4	4(2^*^)	10	60	50	100	50	100
3-Week IM (Zagreb) [25,26]	IM	3	0, 7, 21	2, 1, 1	4	4(2^*^)	7.5	45	47.5	85	47.5	85
1-Month 2-site ID (Updated TRC) [27]	ID	4	0, 3, 7, 28	2, 2, 2, 2	4	0.8	10	60	30	80	25	75
1-Week 2-site ID [28]	ID	3	0, 3, 7	2, 2, 2	3	0.6	7.5	45	22.5	60	22.5	60
1-Week 4-site ID [29–31]		3	0, 3, 7	4, 4, 4	3	1.2–1.5	7.5	45	37.5	75	22.5	60
1-Month simplified 4-site ID [32]	ID	3	0, 7, 28	4, 2, 1	3	0.7	7.5	45	25	62.5	22.5	60

* Calculated assuming use of 0.5 mL vials.

**Table 2:** Costs for calculating the cost-effectiveness of post-exposure vaccination regimens per patient treated. Costs in bold were used as the default in simulations. Insulin syringes/needles were compared to standard 1CC syringes for ID regimens only. Non-medical costs were only considered when examining costs to patients.

Cost	Parameter	Unit cost estimate (USD)	Details
Medical	Material costs per injection (needles, syringes)	**$0.033**–0.4	Standard syringe – 2/consultation for ID regimens, 1/consultation for IM
		0.1455	Insulin needle – 1/consultation
	Overhead per clinic visit (staff salaries & administration)	$0.5–**1.2**	Depends on country/setting
	Vaccine costs per vial	$6.6–20 (**$10**)	Depends on country/setting
Non-medical	Transport and accommodation costs per clinic visit	$2–15	Depends on country/setting
