# Supplementary figures and images for: Modelling to inform prophylaxis regimens to prevent human rabies
Source: Vaccine. Author manuscript; Available in PMC 2022 Feb 16. (PMC7612382; doi:10.1016/j.vaccine.2018.11.010)

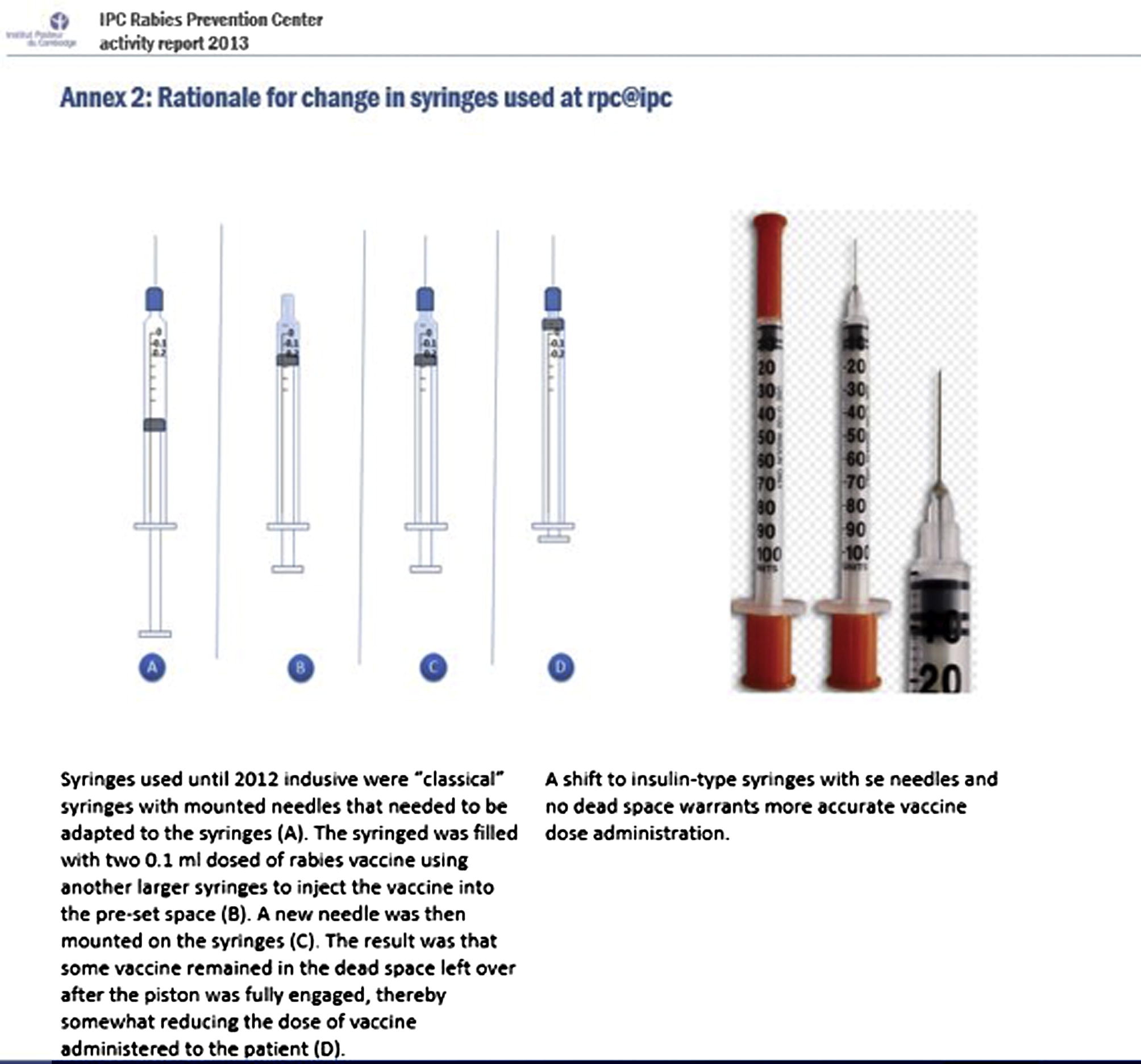

Supplement: Fig S1 [file EMS142107-supplement-Fig_S1.jpg]
